# Event and Comment

**Published:** 1911-03-15

**Authors:** 


### DENTAL REGISTER.

**Vol. LXV.** **March 15, 1911.** **No. 3**

### EVENT AND COMMENT.

MORE INTEREST IN GOOD TEETH. It can hardly be questioned that there is a more widespread interest today in preserving the teeth than ever before. The work of public education is being promoted in almost every city and town of consequence in the country and the general public is becoming more intelligent as to oral hygiene measures. The most promising fact is that the subject of good teeth is being brought to the attention of our school children in a way that ought to make for the future care of the dental health of the nation. As an illustration a school teacher sent a pupil to our office recently for some extracted teeth with a special request that one of them be split open so as to show the pulp canal, as she had been unable to get a specimen of that kind by any means at her command.

When school teachers are so interested to present dental anatomy to their pupils in such a concrete and effective manner, it would seem that the Dental Profession could do no better work than to make some adequate provision for supplying such needed facilities as shall make the teaching of the essential facts of the dental organs practicable. We ought to have some good charts and actual specimens to place at the disposal of every teacher. Would it not be possible for the dentists in every community to provide not only suitable charts and museum specimen, but some one of their number who should prepare a course of demonstrated lectures which could be presented in every school in the community at some favorable time during the year?

We are aware that some work of this kind is and has been, done; but, so far as it has come under our observation the work has not been systematically nor very effectively done. There is need for, not only the man and the apparatus, but for a cooperate effort by the profession and the school authorities, which should be followed up with instruction by the school teacher with simple but effective methods of oral hygiene. Very few school teachers could give this fundamental work as effectively as an intelligent and enthusiastic dentist; and on the other hand, few dentists could so forcibly and effectively instruct young people as a teacher who knows the facts of oral hygiene.

HOPELESS TEETH. The question very often comes to the conscientious dentist as to whether he shall endeavor to save a particularly unpromising tooth or extract it. In these days of the successful handling of cases of peridentitis, even in the badly suppurating stages, enthusiasts are inclined to undertake the almost impossible. A very eminent dental scientist is recently quoted as saying that when an anterior single rooted tooth becomes mobile, it is never cured. This of course is a broad and sweeping statement, and we have no doubt its author would want to qualify it in his practical efforts to preserve the dental arch complete. Such teeth can generally be freed from an active diseased condition and be made useful for many years, and although the peridental structures may never resume their normal state as to form, they can be freed from irritants and infection and be of much more practical benefit to the patient than any form of artificial substitute however well conceived and constructed. In the same journal fom which this quotation is taken the editor relates a per onal experience which he had as a young man with a lower third molar which an older dentist had failed to successfully treat, and which after most painstaking endeavor he was enabled to make comfortable and useful for twenty-five years, much to the great pleasure of his patient. There can be little doubt that many in the profession are finding altogether too many hopeless cases in their practices. The compensations for lost teeth, especially the grinding teeth are not to be found in artificial restorations to the extent that many prosthodontists believe. We must give way to much prosthetic repair endeavor because of accidental and diseased encroachments, but it surely is a mistake to condemn what may be made most useful organs by good treatment. Because we find teeth in such apparently bad pathologic conditions, is no good reason for condemning them to the forceps, our patients deserve first consideration, and if we can't successfully treat these cases we should make it our business to find out how our confieres are handling similar conditions.

